# The Clot Thickens: Differential Coagulotoxic and Cardiotoxic Activities of Anguimorpha Lizard Venoms

**DOI:** 10.3390/toxins16060283

**Published:** 2024-06-20

**Authors:** James Dobson, Abhinandan Chowdhury, Jeremie Tai-A-Pin, Harold van der Ploeg, Amber Gillett, Bryan G. Fry

**Affiliations:** 1 Adaptive Biotoxicology Lab, School of the Environment, University of Queensland, St Lucia, QLD 4072, Australia; j.s.dobson@outlook.com.au (J.D.); abhinandan.choudhury@uq.edu.au (A.C.); 2 Venomenal Volunteers, The Netherlands; jeremie@venomenal.com; 3 Working Group Adder Research Netherlands, RAVON, 6525 ED Nijmegen, The Netherlands; harold@venomenal.com; 4 FaunaVet Wildlife Consultancy, Glass House Mountains, QLD 4518, Australia; drambergillett@hotmail.com

**Keywords:** lizard, venom, evolution, coagulation, *Heloderma*, *Varanus*

## Abstract

Despite their evolutionary novelty, lizard venoms are much less studied in comparison to the intense research on snake venoms. While the venoms of helodermatid lizards have long been assumed to be for defensive purposes, there is increasing evidence of toxic activities more useful for predation than defence (such as paralytic neurotoxicity). This study aimed to ascertain the effects of *Heloderma*, *Lanthanotus*, and *Varanus* lizard venoms on the coagulation and cardiovascular systems. Anticoagulant toxicity was demonstrated for the *Varanus* species studied, with the venoms prolonging clotting times in human and bird plasma due to the destructive cleavage of fibrinogen. In contrast, thromboelastographic analyses on human and bird plasmas in this study demonstrated a procoagulant bioactivity for *Heloderma* venoms. A previous study on *Heloderma* venom using factor-depleted plasmas as a proxy model suggested a procoagulant factor was present that activated either Factor XI or Factor XII, but could not ascertain the precise target. Our activation studies using purified zymogens confirmed FXII activation. Comparisons of neonate and adult *H. exasperatum*, revealed the neonates to be more potent in the ability to activate FXII, being more similar to the venom of the smaller species *H. suspectum* than the adult *H. exasperatum.* This suggests potent FXII activation a basal trait in the genus, present in the small bodied last common ancestor. This also indicates an ontogenetic difference in prey preferences in the larger *Heloderma* species paralleing the change in venom biochemistry. In addition, as birds lack Factor XII, the ability to clot avian plasma suggested an additional procoagulant site of action, which was revealed to be the activation of Factor VII, with *H. horridum* being the most potent. This study also examined the effects upon the cardiovascular system, including the liberation of kinins from kininogen, which contributes to hypotension induction. This form of toxicity was previously described for *Heloderma* venoms, and was revealed in this study was to also be a pathophysiological effect of *Lanthanotus* and *Varanus* venoms. This suggests that this toxic activity was present in the venom of the last common ancestor of the anguimorph lizards, which is consistent with kallikrein enzymes being a shared toxin trait. This study therefore uncovered novel actions of anguimorph lizard venoms, not only contributing to the evolutionary biology body of knowledge but also revealing novel activities to mine for drug design lead compounds.

## 1. Introduction

Despite their evolutionary novelty, lizard venom systems have been the subject of few studies. Even that of the iconic Gila monster (*Heloderma suspectum*), is quite data deficient. Previous work, however, has uncovered a wide diversity of bioactivities acting on diverse physiological systems including cardiovascular, blood coagulation, and neurological functions [1,2,3,4,5,6,7,8,9,10,11,12,13,14,15,16,17,18,19,20,21,22,23]. The diversity of activities is paralleled by complex venoms containing a myriad of toxin types, with the overall venom protein composition and variation within toxin types showing evidence of accelerated duplication and diversification under positive selection pressure [4,5,6,9,24]. Extensive variation within the lizard mandibular venom glands has also been noted [5,9]. The basal state, as present in anguid lizards, is multiple small compartments each containing lumens for storing liquid venom and with drainage channels leading upwards towards the teeth. On two separate occasions, once at the base of *Heloderma* and convergently at the base of the *Lanthanotus*/*Varanus* clade, the glands become dramatically enlarged, accompanied by the fusion of multiple compartments to reduce the number of compartments to six in each case, and with the formation of much larger lumens [4,5,6].

Clinical and anecdotal reports of coagulopathy have been attributed to the bites of varanid and helodermatid lizards. While low fibrinogen and prothrombin levels are occasionally reported post *Heloderma* bite, the hemograms of most patients reveal little blood-related disturbance and rarely manifest as symptoms [25]. In contrast, bites from varanid lizards consistently report profusive bleeding exceeding the blood loss expected from the mechanical damage caused by dentition alone [4,11,26,27]. However, as bites are less medically significant than those of helodermatid lizards, few clinical reports complete with hemograms exist. Anecdotally, profusive bleeding has been attributed to multiple varanid species. Most of these bites occur to herpetologists unwilling to remove the lizard by force and risk harm to the specimen, instead allowing the lizard ample time to chew venom into the wound. In a controversial case of a woman who later succumbed to renal failure and cardiac arrest after a *V. bengalensis* bite, prolonged whole blood clotting times in addition to notable oozing of blood from the wound site nine hours after the incident was experienced [27]. Some (who were not involved directly with the case in any capacity) are sceptical of this account claiming the more likely culprit to be the snake *Daboia russelli* [28]. However, these species are clearly distinguishable, likely leading to the witness (including governmental wildlife authorities) making a positive identification. In addition, the laceration wounds produced by a large-bodied varanid lizard and a puncture wounds of a viper would have been obvious, an inconsistency that clinicians and authors directly involved in the case would have commented on. One of us (BGF) corresponded with the reporting clinicians Vikrant and Verma to enquire about the wound and they indeed confirmed it was not the characteristic pair of puncture wounds one would expect from a *Daboia* bite but was a series of lacerations consistent with a varanid lizard bite. Helodermatid bites can also produce serious symptoms such as angioedema, hypotension, cardiac ischemia, and bronchoconstriction that can be fatal [25,29,30,31,32]. While less serious, similar symptoms of angioedema and faintness due to hypotension have been recorded in bites from *V. griseus* and *V. komodoensis* [33,34,35]. This suggests that varanid lizards may possess coagulotoxic and vasoactive venom components similar to those of *Heloderma* species.

Venoms evolve new toxins whereby ‘leaky expression’ venom glands promiscuously express proteins normally used elsewhere in the body for a normal physiological function [36,37]. For example, the neurotoxic peptides called three finger toxins that are responsible for lethal paralytic effects following envenomations by snakes such as death adders (*Acathophis* species) are a modified version of a peptide normally found in the brain. [38,39]. Subsequent to their recruitment for use as a toxin, such components typically undergo explosive duplication and diversification of gene copies that are selectively expressed in the venom gland. Toxin recruitment can occur at any trophic level. For example, while the blood clotting enzyme factor Xa was recruited for use as a toxin in the last common ancestor of the Australian elapid snake radiation, an additional blood clotting protein (factor Va) was recruited much later, in the last common ancestor of taipans (*Oxyuranus* species) and brown snakes (*Pseudonaja* species) [40,41,42].

Investigations into the composition of Anguimorpha lizards have revealed a wide diversity of components to be present, some with apparent lineage-specific presence, paralleled by an equal diversity of actions upon the cardiovascular system, blood coagulation, and neurological function [4,5,6,9,24,43]. While some toxin types are known only from particular lineages, shared across all the lizard venoms are Group III phospholipase A_2_ and kallikrein-type serine proteases [4,5,6,8,9,11].

The PLA_2_ toxins in lizard venoms are phylogenetically distinct from the forms present in snake venoms [36]. The Group III PLA_2_s isolated from *H. horridum* and *V. varius* have been demonstrated to share platelet aggregation inhibition activity via the same mechanism, producing anticoagulation [4,10]. Similar PLA_2_s have been recovered from the proteomes and transcriptomes of other varanid and helodermatid species [4,5,6,8,11]. Other than activities upon platelets, the inhibitory action of varanid lizard venoms on blood clotting enzymes and factors has yet to be investigated. This is in contrast to the intense research into snake venoms, which has yielded a vast array of toxin types acting on the full spectrum of the blood clotting cascade [39,44,45,46,47,48,49,50,51,52,53,54,55,56,57,58,59,60,61,62,63,64,65,66,67,68,69,70,71,72,73,74,75,76,77,78,79,80,81,82,83,84,85,86,87,88,89,90,91,92,93,94,95,96,97,98,99,100,101,102,103,104,105,106,107,108,109,110,111,112,113,114,115,116,117,118,119,120,121,122,123,124,125,126,127,128,129,130].

In contrast, the kallikrein-type serine protease toxins in lizard venoms form a monophyletic group with snake toxins, representing a toxin type recruited at the base of the Toxicoferan radiation [4,11]. These toxins evolved as exapted forms of salivary kallikreins through increased expression levels. Consequently, these are the most highly expressed toxin type across the Anguimorpha lizards, with subsequent extensive duplication, diversification, and neofunctionalisation [4,5,6,9,11]. Anticoagulant effects of varanid and helodermatid lizard venoms that target the clotting cascade have been attributed to kallikreins producing fibrinogenolytic activity disrupting the final stage of clot production [2,11,23]. This includes extensive variation in the potency of fibrinogenolytic activity with the subgenera *Odatria*, *Varanus*, and *Hapturosaurs* clades, displaying amplification of the trait and suggesting active selection pressures associated with this activity likely linked to prey selection or predator deterrence [2].

A previous study suggested a procoagulant activity for *Heloderma* venoms, but the site of action was not elucidated as the experimental design was limited to using whole plasma and FXI-depleted plasma, thereby it was unable to ascertain if the site of action was FXI activation or an upstream FXII activation site of action [131]. However, the study suggested that there were additional sites of action as *H. suspectum* was still able to clot FXI-depleted venom, suggesting additional zymogens downstream of FXI in the intrinsic pathway, including the common pathway components, or that there was additional action upon the extrinsic pathway.

Hypotension has been shown to be induced by a myriad of toxin types that lower blood pressure due to the relaxation of vascular smooth muscle causing vasodilation. The first documented toxin type with this activity was kallikrein enzymes from *Heloderma* venoms that cleaved kininogen to release the hypotension-inducing bradykinin [1,15]. In addition to vasodilation, the release of bradykinin from kininogen also results in increased vascular permeability leading to angioedema, pain, and bronchoconstriction through the contraction of non-vascular smooth muscle; consistent with symptoms commonly reported in severe *Heloderma* envenomations including angioedema and bronchoconstriction [25,29]. Furthermore, serious bites from *V. griseus* and *V. komodoensis* displayed symptoms similar to *Heloderma* envenomations and consistent with bradykinin release [33,34,35]. However despite the abundance of homologous kallikrein enzymes within varanid lizard and *L. borneensis* venoms [4,5,6,11,132] the bradykinin liberating activity of these venoms has not been investigated.

Despite the potential for extensive variation in venom activities reflective of the diversity represented by the *Heloderma* and *Varanus* genera alone, studies have concentrated on a few species or have only used human models for experiments. While human-derived blood components could represent mammalian models, other taxa plasmas need to be tested if function in prey subjugation or natural predator deterrence is to be discussed. As prior studies focussed on fibrinogen effects [2,11], the effects of varanid lizard venoms on the complete cascade has not yet been investigated. In addition, potential ontogenetic changes in venom activity in lizard venoms have been completely neglected despite being well documented in venomous snakes.

Therefore, this study addresses these knowledge gaps by testing *Heloderma* and *Varanus* species for relative coagulotoxic and cardiotoxic activity. The mechanism of action of varanid venom-induced anticoagulant toxicity was tested using the varanid species *V. scalaris*, *V. prasinus*, and *V. varius*, which represent a tremendous diversity of taxonomy, ecological niche, size, and predatory ecology. Testing was undertaken on both human and avian plasmas. Procoagulant toxicity was also ascertained using human and chicken (*Gallus gallus*) plasma along with isolated human zymogens to test if the procoagulant activity of *Heloderma* venom is due to the action of FXII [131]. As avian plasma lacks FXII [133], any procoagulant activity from *Heloderma* venoms on chicken plasma must be acting elsewhere on the clotting cascade. Ontogenetic changes in *H. exasperatum* procoagulant toxicity were tested using venom from hatchlings and their parents. The kinin-generating activities of *Heloderma*, *Lanthanotus*, and *Varanus* venoms were investigated by ascertaining the ability to cleave kininogen.

## 2. Results

### 2.1. Anticoagulant Effects of Varanus Venoms

**Figure 1:**
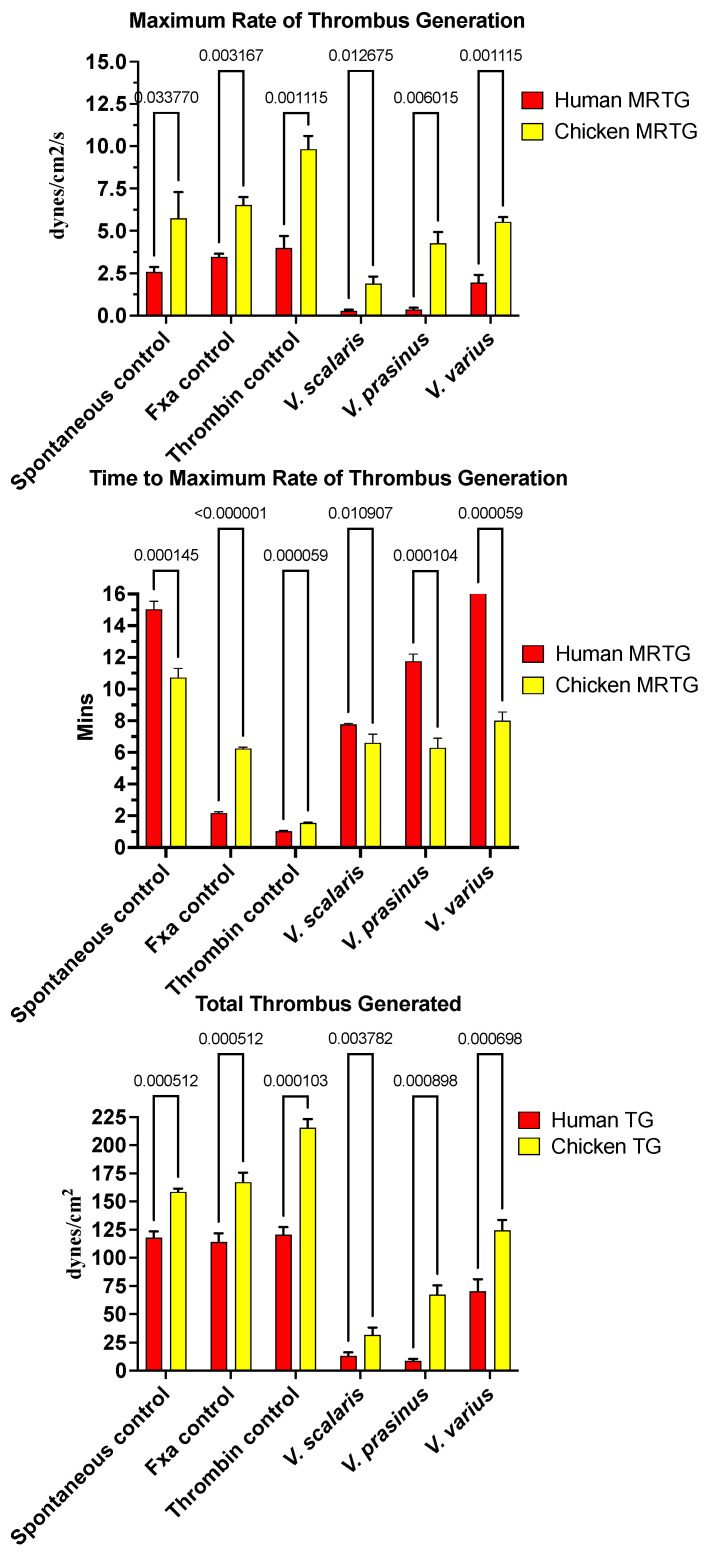
Clot formation kinetic results for varanid lizard species’ venom tested on human (red) and chicken (yellow) plasma relative to controls including: maximum rate of thrombus generation (MRTG), time to maximum rate of thrombus generation (TMRTG), and total thrombus generated (TTG). Experiments were run in triplicate with error bars denoting SE. Statistics are unpaired *t*-tests with Welch correction. *p*-values < 0.05 are considered significant.

Varanid lizard venoms significantly affected the clotting of both the chicken and human plasma (Figure 1). The speed of clot formation (maximum rate of thrombus generation, MRTG) was significantly reduced by all three species against both plasmas, with a rank order of *V. scalaris* > *V. prasinus* > *V. varius* on chicken plasma (*p* < 0.05), and *V. scalaris* = *V. prasinus* > *V. varius* on human plasma (*p* < 0.05). Differential effects upon the rate of clot formation (total thrombus generation, TTG) varied between plasmas with a rank order of *V. scalaris* > *V. prasinus* > *V. varius* (*p* < 0.05), but on human plasma, there was no statistical difference between *V. scalaris* and *V. prasinus* (*p* > 0.05) although both were significantly more potent than *V. varius* (*p* < 0.05). As both the rate of thrombus generation (MRTG) and the total amount of thrombus generated (TGG) were significantly affected, this is indicative of the depletion of fibrinogen levels through destructive cleavage of this fibrin clot precursor.

Intriguingly, for the time to maximum rate of thrombus generation (TMRTG) (Figure 1), *V. prasinus* and *Varanus scalaris* venoms produced statistically similar TMRTG values to the human-derived FXa control on chicken plasma (*p* > 0.05). An exception to this activity was *V. varius* on human plasma, which delayed the maximum clotting rate (Figure 1). While these faster TMRTG values do not match human-derived enzyme control values on human plasma, they are significantly faster than the spontaneous clotting controls (Figure 1). This suggests two competing hypotheses: first that the direct action on fibrinogen is in a pseudo-procoagulant manner, as widely seen in snakes [134], whereby fibrinogen is converted to weak, unstable abnormal clots that rapidly break down, thereby contributing to the net depletion of fibrinogen levels, thus potentiating the anticoagulant effect. The competing hypothesis is that the venoms are activating a clotting factor, thereby accelerating clot formation despite simultaneously reducing fibrinogen levels. The second hypothesis was ruled out because none of the varanid lizard venoms (*V. prasinus*, *V. scalaris*, or *V. varius*) activated any of the plasma zymogens tested (FVII, FIX, FX, FXI, FXII, and prothrombin). Thus, a dual-action effect upon fibrinogen appears to be the most parsimonious explanation, which is again consistent with the effects noted for multifunctional snake venom kallikrein enzymes [135,136], which share a molecular evolutionary history with the varanid lizard venom forms, being a basal Toxicoferan toxin type.

None of the varanid venoms tested inhibited any of the activated clotting enzymes tested (XIIa, IXa, XIa, Xa, or IIa (thrombin).

### 2.2. Procoagulant Effects of Heloderma Venoms

**Figure 2:**
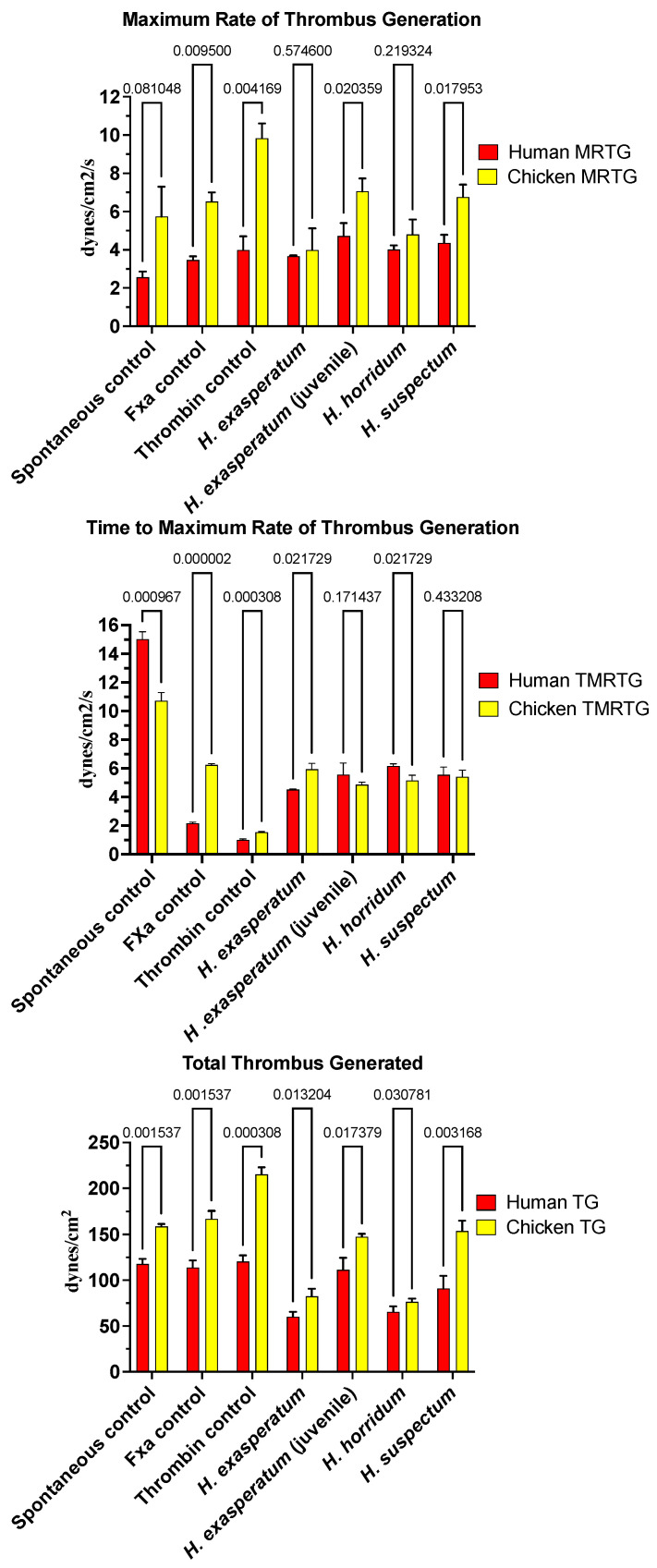
Clot formation kinetic results for helodermatid lizard species’ venom tested on human (red) and chicken (yellow) plasma relative to controls including: maximum rate of thrombus generation (MRTG), time to maximum rate of thrombus generation (TMRTG), and total thrombus generated (TTG). Experiments were run in triplicate with error bars denoting SE. Statistics are unpaired *t*-tests with Welch correction. *p*-values < 0.05 are considered significant.

In contrast to the anticoagulant effects of the varanid venoms, *Heloderma* venoms produced procoagulant activity on both human and chicken plasmas (Figure 2). The time to maximum rate of thrombus generation (TMRTG) was accelerated relative to the spontaneous clotting control for all *Heloderma* venoms tested against both plasmas. For the chicken plasma, the rate was nearly identical to that of the FXa control. There were variations between the different *Heloderma* venoms in the total thrombus generated (TTG). For the TTG on avian plasma, the adult *H. suspectum* and neonate *H. exasperatum* venom equipotently produced stronger clots than the adult *H. exasperatum* adult venom and adult *H. horridum*, which were in turn equipotent to each other (Figure 2) (*p* > 0.05). This is indicative of a stronger procoagulant effect for the adult *H. suspectum* and neonate *H. exasperatum* relative to the adult *H. exasperatum* and *H. horridum*; the effects of the latter, while not as potent, were still not trivial. On the human plasma, while adult *H. suspectum* and neonate *H. exasperatum* venom were again more potent in the total amount of thrombus generated, the difference with the adult *H. exasperatum* adult venom and adult *H. horridum* was less than that seen for avian plasma (Figure 2). While the total clot formation differed between the venoms across the two plasma types, the maximum rate of thrombus generation (MRTG) did not vary. This suggests that the enzymes have equipotent rates of action, and therefore, the differences in clot strength may be due to isoformic variations.

**Figure 3:**
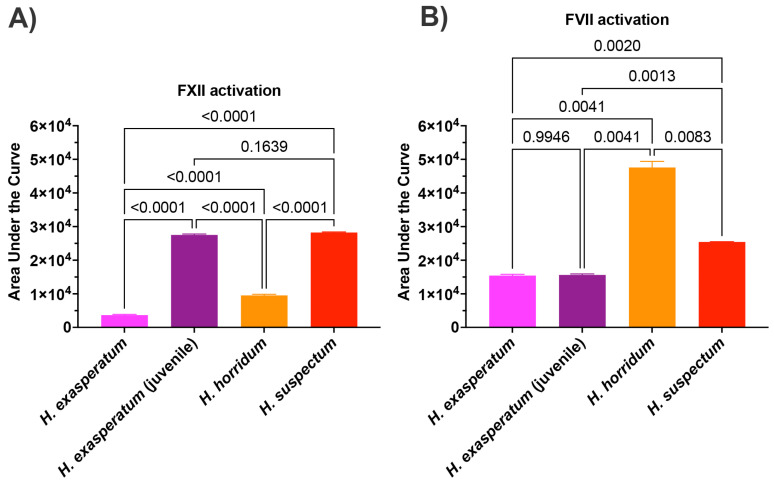
(**A**) Area under the curve (AUC) for *Heloderma* venom activation of (**A**) Factor XII and (**B**) Factor VII. Experiments run in triplicate with error bars denoting SE. Statistics are Brown–Forsythe and Welch ANOVA tests. *p*-values < 0.05 are considered significant.

In order to ascertain the underlying differences in biochemistry, tests were undertaken for activation of the zymogens of FVII, FIX, FX, FXI, FXII, and prothrombin to ascertain the site of procoagulation. Fluorescent substrate assays demonstrated FXII activating activity for all *Heloderma* venoms tested (Figure 3A). Consistent with the thromboelastography results, adult *H. suspectum* and neonate *H. exasperatum* venoms were nearly equipotent in their FXII zymogen activation levels, and more potent relative to the adult *H. exasperatum* and *H. horridum* venoms (Figure 3B). FVII was another zymogen shown to be activated by *Heloderma* venoms. However, in this case, while the adult *H. suspectum* and neonate *H. exasperatum* venoms were again equipotent, they were not the most potent, with the adult *H. horridum* being dramatically more potent than the other venoms.

**Figure 4:**
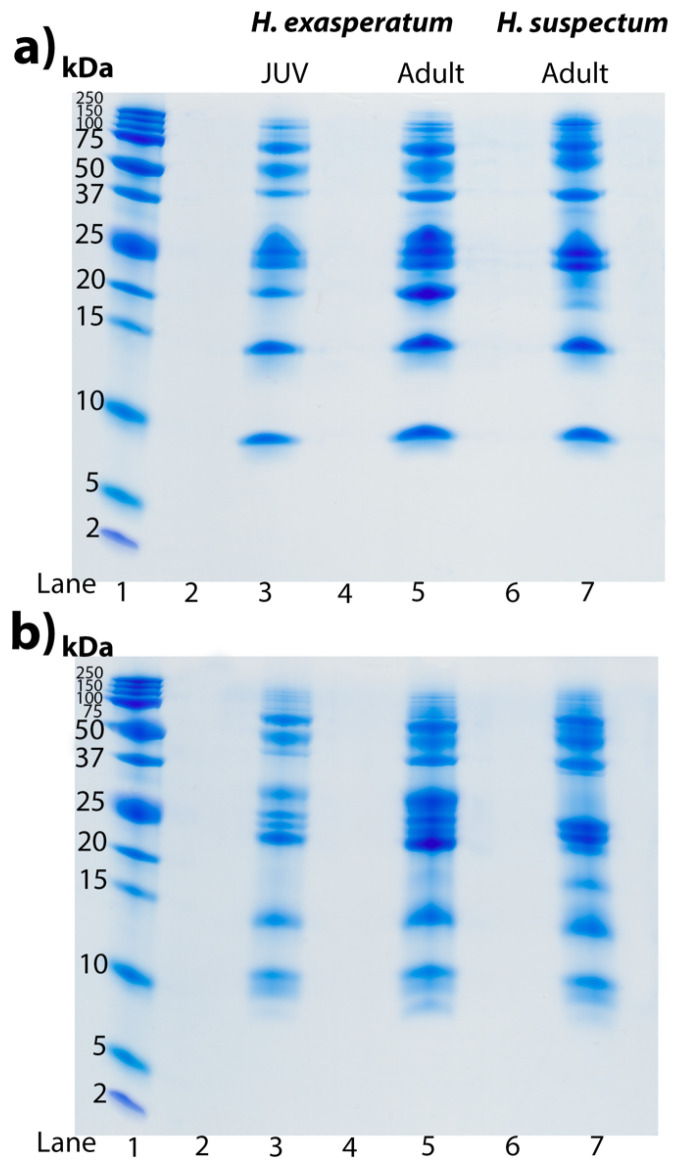
Tris-tricine SDS PAGE gels displaying profiles of *H. exasperatum* juvenile (JUV, lane 3), *H. exasperatum* adult (lane 5), and *H. suspectum* (lane 7) venoms (25 μg each) under (**a**) non-reduced conditions and (**b**) reduced conditions. Lane 1 contains standard molecular weight ladder reference with bands labelled in kilodalton (kDa). Lanes 2,4, and 6 were left blank as spacers. Gels were stained with Coomassie blue.

Tris-tricine gels revealed identical venom profiles of the *H. exasperatum* neonate and adult, particularly around the expected resolving range of kallikreins (30–50 kDa) [11] (Figure 4). This suggests the differences in the venom activities are not due to the differences in the relative abundance toxin types, but rather ontogenetic differences in the presence of particular isoforms within a toxin type, as has been noted before in snake venom, with *Daboia* venoms varying in isoforms rather than quantity as a reflection of age [135,136]. These gel results provide a testable hypothesis to be investigated in future transcriptomics work.

### 2.3. Cardiovascular System Effects of Heloderma and Varanus Venoms

**Figure 5:**
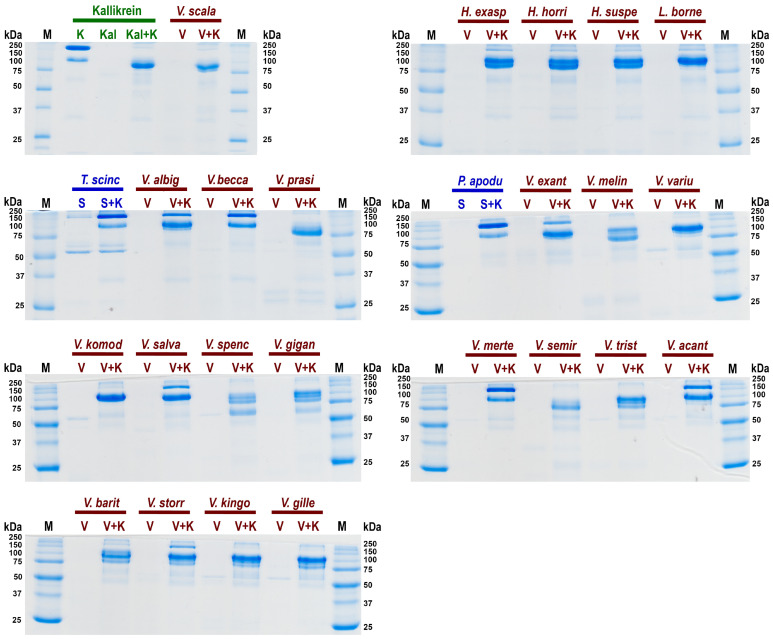
SDS PAGE gels of relative cleavage of kininogen (K) by: in green the positive control kallikrein (Kal); in blue the negative control saliva (S) species *Pseudopus apodus* and *Tiliqua scincoides* in blue; and in red the anguimorph lizard venoms (V) species *Heloderma* *exasperatum*, *H. horridum*, *H. suspectum*, *L. borneensis*, *Varanus acanthurus*, *V. albigularis*, *V. baritji*, *V. beccarii*, *V. exanthematicus*, *V. giganteus*, *V. gilleni*, *V. kingorum*, *V. komodoensis*, *V. melinus*, *V. mertensi*, *V. prasinus*, *V. salvadorii*, *V. scalaris*, *V. semiremex*, *V. spenceri*, *V. storri*, *V. tristis*, and *V. varius*. First and last lanes contain ladder controls with molecular weights shown alongside corresponding marker (M) bands in kilodaltons (kDa). Gels stained with Coomassie blue.

A further cardiovascular system investigation was undertaken to ascertain the relative effect of kininogen cleavage by the venom kallikrein-type serine protease enzymes to liberate the hypotension-inducing low-molecular weight kinins such as bradykinin (Figure 5). The negative control species (*P. apodus* and *T. scincoides*) salivary secretions do not appear to cleave kininogen to generate kinins. However the venom of the varanoid species displayed generation of degradation products consistent with the plasma kallikrein control and *Heloderma* venoms (which are known to release kinins from kininogen). The greatest level of kininogen cleavage (disappearance of all or most of the high molecular weight kininogen) during the experimental time period was observed for *H. exasperatum*, *H. horridum*, *H. suspectum*, *L. borneensis*, *V. baritji*, *V. exanthematicus*, *V. gilleni*, *V. kingorum*, *V. komodoensis*, *V. giganteus*, *V. melinus*, *V. prasinus*, *V. salvadorii*, *V. scalaris*, *V. semiremix*, *V. spenceri*, *V. storri*, *V. tristis*, and *V. varius*. Lower levels of cleavge within the experimental time period was observed for *V. acanthurus*, *V. albigularis*, *V. beccarii*, and *V. mertensi*. 

Consistent with extensive kallikrein sequence and expression diversity observed in anguimorph lizard venoms [5,11], there was corresponding diversity in cleavage of kininogen and products formed (Figure 5). Some, such as *V. scalaris*, produced band patterns virtually identical to that of the kallikrein control, while others such as *V. spenseri* produced additional degradation products. Future work is necessary to ascertain the functional implications of this cleavage diversity. This suggests that the varanid venoms have evolved to cleave kininogen at multiple sites. This provides a solid platform for future work to map the sites of cleavage and ascertain the length and the relative hypotensive bioactivity of the liberated kinins. Future work should also investigate the relative rate of activity. Venom kallikreins are exapted versions of salivary kallikreins, with these enzymes providing the starting substrate for the weaponised venom versions [36,137]. 

Thus, this work provides the first evidence for widespread kininogen cleavage, while also providing evidence for differential cleavage, and lays the groundwork for future investigations. As such, this provides a platform for future work testing both time series to ascertain relative rates of activity, and also using differential masses to account for gland size. Future work should include in vivo work with purified enzymes to ascertain their role in previous hypotensive activity such as for *V. komodoensis* [6].

## 3. Discussion

This study revealed a striking dichotomic phenotypic pattern, with *Varanus* venoms exhibiting anticoagulant activity mediated by the direct depletion of fibrinogen levels, while conversely, *Heloderma* venoms showed procoagulant activity through the activation of Factor XII and Factor VII. This dichotomy parallels the two lineages independently evolving complex mandibular venom glands out of the ancestral state seen in anguid lizards [4,5,6].

### 3.1. Varanus Venom Anticoagulation

Varanid lizard venoms have previously been shown to produce potent fibrinogenolytic activity, which produces anticoagulation by depleting fibrinogen levels [2,11]. Here, representatives of three clades that have amplified the fibrinogenolytic trait were tested on mammalian and avian plasmas displaying considerable variability in their ability to reduce clotting in different taxon plasma types (Figure 1). Congruent with previous research [2], *V. prasinus* and *V. scalaris* were more potent on mammalian plasma than *V. varius*, and were also more potent on avian plasma (Figure 1). These species are both small arboreal lizards with birds and mammals contained within their diets, suggesting the presence of selection forces due to the risk of high prey escape [2].

*V. prasinus* and *V. scalaris* displayed reduced time to maximum rate of thrombus generation (TMRTG) times faster than spontaneous clot formation, suggestive of a pseudo-procoagulant direct action upon fibrinogen, whereby weak, unstable fibrin clots rapidly break down, thereby contributing to the net levels of fibrinogen depletion. Such an activity has been well documented as being widespread in snake venoms [135,136,138,139,140]. Consistent with these venoms being pseudo-procoagulant rather than truly procoagulant, neither venom activated blood clotting enzyme zymogens. 

*Varanus varius* is a large-bodied generalist predator consuming a wide variety of prey items [141,142]. While the diet of these large predators incorporated more mammals and birds than the smaller species tested, the effect on plasma types was minimal (Figure 1). However, the coagulotoxic effect of PLA_2_-mediated platelet blocking activity has been documented for this species’ venom, and hypotension, smooth muscle contraction, and ion channel binding have also been demonstrated [4]. Indeed, the PLA_2_ activity of *V. varius* far exceeds other species, suggesting a strong contribution by PLA_2_-driven activities such as platelet inhibition to subjugate prey for this species and the related *V. komodoensis* [4,11].

### 3.2. Heloderma Venoms’ Procoagulation

Previous work using FXI-depleted plasma suggested that *Heloderma* venom activated either FXI or FXII [131]. However, in that prior study no specific tests were undertaken to ascertain the site of action. Assays were restricted to factor depleted plasma studies, with the procoagulant activity disappearing when FXI-depleted plasma was tested, which left both FXI and FXII as potential pathophysiological targets. However, using purified zymogens, the data in the current study revealed that the venoms in fact activated FXII, with no activation of FXI. Further to the revelation that *Heloderma* venoms activate FXII, we showed the activation of FVII as another procoagulant toxic action. All our *Heloderma* venoms displayed significant procoagulant potency. However, there was a strong ontogenetic signal whereby the adults of the small species *H. suspectum* and neonate of the large species *H. exasperatum* were more potent activators of FXII than the adults of *H. exasperatum* and also adults of the large species *H. horridum.* Conversely, the adult *H. horridum* was a significantly more potent activator of FVII. Future work using purified enzymes is required to ascertain the enzyme class responsible for the activation of the FXII and FVII zymogens to produce the procoagulant toxicity, but the abundant kallikrein enzymes are the most likely responsible toxin type.

From an evolutionary perspective, these results suggest two competing hypotheses in regards to morphological size and venom phenotype, both of which take into account that *H. suspectum* is the most basally split species among the extant *Heloderma* [143]. First, if *H. suspectum* represents the basal morphotype, and with the beaded lizards thus representing a secondary evolution of gigantism within the genus, then this suggest that FXII activation is the basal procoagulant condition which is retained in the neonates of the larger species. This also suggests downregulation of FXII activation in the adult venom phenotype of the non-*suspectum* species, with an ontogenetic change towards a less FXII-activating venom and a more FVII-activating venom. Under this hypothesis, the *H. suspectum* venom phenotype represents the ancestral state that is retained through all life stages for this species, and changes only in the larger species as they increase in size into adulthood. Such a scenario has been observed in the *Bitis* genus of viperid snakes where the basal condition is small snakes with procoagulant venoms, which has been derived on two independent occasions into giant species with anticoagulant venoms [144,145]. The competing hypothesis is that the basal morphological condition of the clade of extant *Heloderma* is that of a large lizard that has an ontogenetic variation in its venom biochemistry, changing from FXII-activating to FVII-activating as it transitions from neonates to adults. In which case, *H. suspectum* represents a case of paedomorphism, whereby they secondarily evolved as a small species and retained the juvenile venom phenotype. Such a scenario has been documented in snakes, such as the paedomorphism observed in the *Pseudonaja* genus of elapid snakes, whereby *P. modesta* has secondarily evolved to be much smaller than all other brown snakes and retains the juvenile venom condition of neurotoxicity, not changing to a procoagulant venom in adulthood as all other *Pseudonaja* species do [146,147]. *P. modesta*, however, is not a basal split, but a highly derived species nested within a clade of large snakes. In the case of the *Heloderma* species, the data favours the first scenario, whereby *H. suspectum* represents the basal state morphologically and in venom biochemistry, consistent with it being the most basally split species.

*Heloderma* venoms are well characterised as being intensely painful, which has led to the long-held belief that their venom evolved solely for a defensive role [148]. Defensive venoms are typically associated with painful cytotoxic or pain-pathway triggering neurotoxic effects, while coagulopathy is a trait associated with predatory venoms [3]. Thus, the procoagulant toxicity revealed in this study and the previous documentation of paralytic neurotoxicity [3] are two venom traits associated with predation rather than defence. Thus, this not only suggests a dual role (defence and predation) for the venom but also that the ontogenetic changes evident in *H. exasperatum*, and the sharing of the same venom phenotype between adult *H. suspectum* and neonate *H. exasperatum*, indicate evolutionary shaping pressure from different prey types between small and large *Heloderma* specimens. Ontogenetic shifts in venom activity are well documented in snakes where the primary prey has changed from juvenile to adult [146,147,149,150,151]. The predatory ecology of *Heloderma* species is data deficient at all life stages, with most of the data acquired during the short-seasonal above-ground period when ground-nesting bird nests are raided for eggs [148]. With *H. suspectum* a semi-fossorial rodent feeder other parts of the year. The suggestion that the fossorial/terrestrial *H. suspectum* consumes less avian prey than the semi-arboreal *H. exasperatum/horridum* [152,153] is consistent with our results revealing the higher activation of FXII by *H. suspectum*, a factor lacking in avian plasma. Conversely, and consistent with the known dietary variation which includes avian prey, *H. horridum* was found to be the most potent activator of FVII of the extrinsic pathway, a pathway shared by mammals and birds [133]. Similarly, *H. horridum* was found to be more paralytically potent on avian nerve–muscle preparations than other *Heloderma* species [3]. Presynaptic neurotoxicity has been shown for both *Heloderma* and *Varanus* venoms, with sites of action including the S3–S4 extracellular loops of the calcium channel Ca_V_1.2 and the sodium channel Na_V_1.4 [3]. While the helofensin toxin type known currently only from *Heloderma* venom has been characterised as being neurotoxic [154], it remains to be elucidated which of the neurotoxic actions in *Heloderma* venoms it is responsible for. In regard to *Varanus* venoms, it remains to be ascertained which toxins are responsible for the observed neurotoxic effects. Thus, the variation in venom biochemistry relative to size and the correlation with prey preference is a rich area of future research involving natural history observations to ascertain prey preference relative to lizard size.

Consistent with previous findings [8,43], the *Heloderma* venoms displayed little variation when analysed by gel electrophoresis. Such a form of venom comparison of course is only at the highest level of venom variation, of gross level changes between toxin classes. It cannot discern between toxin isoforms present within the bands. However, the similarity in the overall venom profile but differential activity suggests variations in toxin isoforms between adult *H. suspectum* and neonate *H. exasperatum* and adult *H. exasperatum* and *H. horridum.* This hypothesis can be tested in future transcriptomic work that sequences the relatively expressed toxins and compares them using molecular phylogenetic methods. Such a phenomenon has previously been documented in snakes, such as within *Daboia* where the same biochemistry was present across each age group, but the neonates were more potent on amphibians but equipotent with adults on mammals [155], suggestive of differential toxin isoform expression within a toxin class rather than the expression of different toxin classes.

### 3.3. Kininogen Cleavage by Heloderma and Varanoid Venoms

In this study, kininogen cleavage was shown to be a widespread activity across the anguimorph venom studied. The results of this study suggest that kininogenolytic activity has been amplified on several occasions, such as for members of the *Odatria* subgenus (*V. gilleni*, *V. kingorum*, *V. scalaris*, *V. semiremex*, *V. tristis*), the *Hapturosaurus* subgenus (*V. melinus*, *V. prasinus*), and the *Varanus* subgenus (*V. komodoensis*, *V. varius*). There was also extensive variation in cleavage sites, consistent with evidence of accelerated gene duplication and diversification of catalytic residues in varanid kallikreins [8,43]. Such dynamic variation extended to sister species. Despite *V. prasinus* completely cleaving kininogen, its sister species *V. beccarii* (which diverged ~2 million years ago) does not [156]. This result contrasts with previous data of the venoms on fibrinogen demonstrating equipotent activity [2], suggesting *V. prasinus* venom possesses kininogenolytic kallikrein isoforms that *V. beccarii* lacks. This extreme variability between closely related species reinforces a functionally diversifying venom system under selection pressure within the varanids.

The documentation of kininogenolytic activity in this study is also consistent with the effects of bite reports. Bradykinin release offers an explanation for the symptoms of faintness, angioedema, and pain experienced by bite victims of *V. griseus* [34,35]. While *V. grisius* was not tested in this study, those documented bite effects are consistent with at least some contribution by kinin release from kininogen, which is further corroborated by two members of the same Afro-Middle Eastern clade (*V. albigularis* and *V. exanthematicus*) in this study possessing kininogenolytic activity (Figure 5). Future work with *V. griseus* venom is of course required to confirm the contribution of kinin release to observed human bite effects, and to investigate the natural history role. The documented hypotension produced by *V. komodoensis* [6], however, is consistent with the kininogen cleavage activity of this study (Figure 5).

The basal varanoid lizard *Lanthanotus borneensis* has previously been shown to possess an enlarged mandibular venom gland homologous to that of the sister genus *Varanus*, and to possess a venom rich in kallikrein enzymes [5,132]. Consistent with this, the venom has previously been shown to be fibrinogenolytic. Regardless, a recent study rejected the classification of *L. borneensis* as venomous [132], with the authors stating kinin-releasing kallikrein activity needed to be present for *L. borneensis* to be considered as venomous, justifying this through the citation of their previous work characterising kininogen cleavage by *Heloderma* venoms [15]. Thus, the homology with *Heloderma* and *Varanus* of gland morphology, fibrinogenolytic activity, and kininogenolytic activity certainly supports the inclusion of *L. borneensis* as a venomous member of the Anguimorpha clade within the Toxicofera.

The kininogenolytic results in this study thus lay a solid foundation for future studies to ascertain the differential sites of cleavage, and what impact these have on the liberated peptides, including the potential for prey selective effects. These results add to the body of knowledge regarding hypotension-inducing effects by lizard venoms. Hypotension has also been shown to be exerted by other peptide toxin types acting upon vascular smooth muscle. B-type natriuretic peptides (BNPs) are ubiquitous across Anguimorpha venoms, being recruited for use as a toxin in the last common ancestor of this clade, and are phylogenetically distinct from the C-type natriuretic peptides recruited into snake venoms [5,6,24,64,157,158,159]. Subsequently, in the last common ancestor of the anguid lizards, extensive mutation occurred in the propeptide region to de novo evolve an additional hypotensive toxin class known as helokinestatins, that are post-translationally liberated, with the natriuretic gene now producing multiple discrete products [5,9,24,160,161]. This remarkable evolution of a multi-product gene is paralleled by the C-type natriuretic peptide gene in snakes, where the propeptide region has also been hypermutated to form multiple new post-translationally liberated toxin types, ranging from hypotensives to neurotoxins. Exendins, which are weaponised versions of the vasoactive intestinal peptides shown to reduce blood pressure and relax femoral arteries in rat and dog models, are currently known only from *Heloderma* venoms [24,162,163,164]. Goannatyrotoxin, currently known only from the varanid lizard *V. glauerti*, is a weaponised version of the pancreatic hormone peptide YY and produces a potent biphasic effect, characterised first by a sharp hypertension stage, followed by sustained hypotension [5]. Celestoxin, currently known only from the anguid lizard *Celestus warren*, also induces hypotension mediated by the relaxation of vascular smooth muscle [5]. Cholecystoxin, currently known only from the varanid lizard *V. varius*, is a remarkable case where a single post-translational modification (a sulfur group added to a tyrosine) is responsible for the bioactivity, with structure–activity relationship studies showing that synthetic peptides made without the tyrosine being sulphated were completely inactive [5].

## 4. Conclusions

The primary findings of this study reveal extensive variation in anguimorph lizard venom activities on avian versus mammalian plasma. The procoagulant activity of *Heloderma* venom was shown to be due to activation of FXII and FVII, not FXI. A fascinating ontogenetic shift in venom activity in lizards has been shown for the first time, revealing *H. exasperatum* juvenile venom shares activity features with that of the smallest *Heloderma* species, *H. suspectum*, suggesting competing hypotheses of basal activity versus paedomorphism. Varanid lizard venoms show extensive variation in their anticoagulant activity on avian and mammalian plasmas, expanding the demonstrable fibrinogenolytic activity of these venoms to whole plasma within biologically relevant experimental times. In addition, while future work is still required, this study provides the first evidence for kinin-generating activity in lizards outside the *Heloderma* genus, including many varanids and *L. borneensis*.

Factor FXII- and FVII-activating activity in *Heloderma* venoms and kinin-generating activity in *Varanus* and *Lanthanotus* venoms are exciting new toxic activities for these genera. While varanid lizards lack certain toxin families that *Heloderma* lizards possess and vice versa, recent studies are uncovering considerable overlap in the activities and physiological targets of their venoms, reflective of the shared evolutionary origin of their venom systems. [2,3,4,5,6,8,9,11,24]. The kallikrein-dominated venoms of these genera display taxon-specific effects, with evidence of a predatory role. Furthermore, the extreme variation in kallikrein-like activity among the Anguimorpha clade demonstrates diversification due to active selection pressures. This work points out many avenues for further research in the hope that research groups will be stimulated into researching the underappreciated venomous lineages that are within Anguimorpha. Kallikrein homologs in snake venoms have been exploited for therapeutic use in the treatment of cardiac and circulatory conditions such as stroke, heart attack, and deep vein thrombosis [165], further emphasising lizard venom kallikreins as having potential for biodiscovery. Furthermore, many toxin families unique to the Anguimorpha are yet to be fully characterised, meaning they may be harbouring intriguing new bioactivities comparable to FXII and FVII activation. 

The implications of this study extend to evolutionary theory about the divergent and convergent paths that venomous lizards and snakes have taken since separating from their last common ancestor [4]. Both FVII (characterised from the *Oxyuranus*/*Pseudonaja* clade of Australian elapids, the natricine snake *Rhabdophis subminiatus*, and vipeid snake *Porthodium volcanicum*) and FXII activating (characterised from the natricine snake *Rhabdophis subminiatus*, and vipeid snake *Porthodium volcanicum*) modes of action have been previously described in snake venoms through the utilisation of distinct toxin types, and with FVII activation evolving on at least two separate occasions within snake venoms [146,166,167,168]. As such, the current study broadly contributes to our understanding of Toxicofera reptile venoms. 

## 5. Materials and Methods

### 5.1. Sample Acquisition and Stocks

All work was undertaken under animal ethics approval, with samples collected under University of Melbourne approval UM0706247 approved 2 May 2004 and University of Queensland approval SBS/403/16 approved 4 July 2016 and biosafety approval (IBSC approval #IBC134BSBS2015, 1 October 2015). Species studied included *Tiliqua scincoides* (captive specimen, unknown founding locality), *Varanus acanthurus* (Newman, WA, Australia), *V. baritji* (Adelaide River, NT, Australia), *V. giganteus* (Sandstone, WA, Australia), *V. gilleni* (captive specimen, Alice Springs, NT, Australia founding locality), *V. kingorum* (captive specimen, Turkey Creek, WA, Australia founding locality), *V. komodoensis* (Singapore Zoo, captive specimen), *V. mertensi* (captive specimen, Kununurra, WA, Australia founding locality), *V. prasinus* (captive specimen, unknown founding locality), *V. scalaris* (Kununurra, WA, Australia), *V. semiremex* (captive specimen, Cairns area, QLD, Australia founding locality), *V. spenceri* (captive specimen, Barkley Tableland, NT, Australia founding locality), *V. storri* (captive specimen, unknown founding locality), *V. trisitis* (captive specimen, Western QLD, Australia founding locality), and *V. varius* (Mallacoota, VIC, Australia), Non-Australian venoms were supplied by licensed biotechnology supply company Alpha-biotoxine Laboratory, Montroel-au-bois, Belgium: *Heloderma exasperatum* (×2 adults pooled), *H. exasperatum* hatchling (×3 individuals milked at 4 months old pooled), *H. horridum*, *H. suspectum*, *Lanthanotus borneensis*, *Pseudopus apodus*, *V. albigularis*, *V. beccari*, *V. exanthematicus*, *V. melinus*, and *V. salvadorii.* Venom was weighed using a XPR2U microbalance. Working stocks of reconstituted venom were made in a 1:1 ratio of deionised water and glycerol (>99.9%, Sigma-Aldrich) at a concentration of 1 mg/mL.

### 5.2. Plasma

Experiments were performed on 3.2% sodium citrated human plasma collected from healthy human donors donated by the Australian Red Cross (Research Agreement #18-03QLD-09, University of Queensland Human Ethics Committee Approval #2016000256). Human plasma was thawed at 37 °C, aliquoted into 1.2 mL quantities, flash frozen in liquid nitrogen, and stored at –80 °C until required. Adult chickens were bled (University of Queensland Animal Ethics approval SBS/020/15/ARC) and collected into 1 mL tubes containing sodium citrate to a total volume of 3.2% sodium citrate. After inverting the tubes for 10 s, samples were centrifuged at 2000 RCF for 10 min followed by removal of the supernatant to a new tube and addition centrifuging at 10,000 RCF for a further 10 min. Once this second supernatant was collected, the plasma was flash frozen in LN2. When required, human and chicken plasma aliquots were rapidly thawed at 37 °C in a Thermo Haake ARCTIC immersion bath circulator (SC150-A40) and immediately used for experimentation.

### 5.3. Thromboelastography

The ability of Anguimorpha lizard venoms to clot or reduce the clotting ability of plasma was measured using a Thromboelastograph^®^ 5000 Haemostasis analyser (Haemonetics Australia Pty Ltd., North Ryde, Sydney 2113, Australia). Natural pins and cups (Lot# HMO3163, Haemonetics Australia Pty Ltd., North Ryde, Sydney 2113, Australia) were used to maintain the same stoichiometry as for the clotting time tests (see Section 5.5. Volumes were proportionally changed to accommodate the larger reaction volume: 7 μL of the 10 mg/mL venom working stock (lyophilised venom in 50% glycerol/50% deionised water) or 7 μL 50% glycerol/50% deionised water for spontaneous controls or 7 μL FXa (Stago catalogue #253047 Liquid Anti-Xa) for the common pathway control or 7 μL of thrombin (stable thrombin from Stago Liquid Fib kit, unknown concentration from supplier (Stago Cat#00673 Liquid Fib)) to produce an immediate clot control, 72 μL CaCl_2_ (25 mM stock solution Stago Cat# 00367 STA), 72 μL phospholipid (solubilized in Owren Koller Buffer adapted from STA C·K Prest standard kit, Stago Cat# 00597), and 20 μL Owren Koller Buffer (Stago Cat# 00360) was combined with 189 μL blood plasma, pipette mixed, and incubated for 30 min at 37 °C. All experiments were run in triplicate.

### 5.4. Coagulation Factor Inhibition

To investigate if *Heloderma* or *Varanus* lizard venoms produce anticoagulation via the inhibition of plasma factors, plasma coagulation assays were carried out on a Stago STA-R Max coagulation analyser (Stago, Asnières sur Seine, France) using active human-derived plasma factors as previously described [125,126,145]. Species included in these assays were: *H. exasperatum*, *H. horridum*, *H. suspectum*, *V. acanthurus*, *V. komodoensis*, *V. mitchelli*, *V. prasinus*, *V. scalaris*, and *V. varius*. The experiments details described in Table 1 were carried out in triplicate. Cofactors calcium and phospholipid were included in the coagulation assays. Controls were conducted with stocks of 50% deionised water and 50% glycerol used in the replacement of venom to ascertain healthy plasma clotting times activated by each factor. Individual factors of the clotting cascade were incubated with the sample venom for 120 s prior to plasma addition as the incubation step allows venom to bind and inhibit its target. All experiments were run in triplicate.

### 5.5. Clotting Factor Activation Assays

To determine if the procoagulant activities of *Heloderma* venoms were due to the activation of Factor XII or other plasma factors, a Fluoroskan Ascent™ (Thermo Scientific, Vantaa, Finland) and 384-well plates (black, lot#1171125, Nunc™ Thermo Scientific, Rochester, NY, USA) were used to measure zymogen activation via the cleavage of a fluorescent substrate. Table 2 details the conditions for each assay. The table denotes manual additions to plate wells with each plate consisting of blanks, zymogen controls, activated factor control, venom controls, and venom + zymogen experiments. Each control or treatment was run in triplicate. Wells contained a total of 0.5 μg of venom and 0.5 μg of zymogen per well both added separately diluted in 10 μL enzyme running buffer without calcium (150 mM NaCl, and 50 mM Tri-HCl (pH 7.3) in treatment groups, while in venom control groups, zymogens were replaced by buffer. Where possible, the activated plasma factors (0.5 μg) were used as positive controls. As FXIIa was not available, 10 μL of kaolin (5 mg of kaolin/mL) was used to convert FXII into FXIIa. Subsequently, automatic pipetting was used to start the reaction by dispensing 70 μL of buffer (5 mM CaCl2,150 mM NaCl, and 50 mM Tri-HCl [pH 7.3]) and Fluorogenic Peptide Substrate ES011 (Boc-Val-Pro-Arg-AMC. Boc: t-Butyloxycarbonyl; 7-Amino-4-methylcoumarin; R&D systems, catalogue# ES011, Minneapolis, Minnesota) in a 500:1 ratio. Plates were run at 37 °C and shaken for three seconds before each measurement. Fluorescence levels were measured each minute for 300 min using the conditions of an excitation wavelength of 390 nm and emission wavelength of 460 nm. The resulting values obtained for blank conditions were subtracted from reactions from the same plate. In addition, as *Heloderma* venoms act directly on the serine protease substrate, thus artificially increasing the fluorescence values, venom on substrate activity alone was subtracted from data obtained from wells containing venom incubated with zymogens. Experiments were also performed with *V. varius*, *V. scalaris*, and *V. prasinus* venoms.

### 5.6. Tricine Gels

In order to compare venom profiles between the *H. exasperatum* hatchlings and adults, 1D gradient gels were run under both reducing and non-reducing conditions using the manufacturer’s (Bio-Rad, Brisbane, Australia) protocol. Twenty-five µg of venom was reconstituted in Tricine loading buffer (Bio-Rad, Brisbane, Australia) with 10 mM DTT added to provide reduced conditions. Reduced samples were incubated at 100 °C for 4 min and 16% Tricine gels were purchased from Bio-Rad (#4563063) as well as the running buffer used (Bio-Rad 10× tris/tricine/SDS running buffer #1610744). The gels were run at 120 V for at room temperature. Gels were stained overnight with colloidal Coomassie brilliant blue G250 (34% methanol, 3% phosphoric acid, 170 g/L ammonium sulphate, 1 g/L Coomassie blue G250). Post staining, gels were de-stained using ultrapure water (PURELAB Flex 2, Brisbane, Australia).

### 5.7. Kininogen Gels

Kininogen cleavage was investigated via 12% SDS PAGE. While BK at ~1 kDa will not be detected, the heavy chain degradation product should indicate BK release as previously described [134]. Freeze dried venom samples were reconstituted in Owren Koller Buffer (STA C·K Prest standard kit, Stago Cat# 00597). Five µg of human HMW kininogen (Sigma-Aldrich, MO, USA Lot# 3F23L73000) and 0.5 µg of venom, or 0.5 µg human HMW plasma kallikrein (Sigma-Aldrich, MO, USA Lot# SLCF5481) for the positive control, were added to a 0.2 mL tube at a total volume of 7 µL and incubated at 37 °C for 30 min. As kinin release is time dependent, 30 min was determined to be long enough to display activity while remaining in the realm of functional relevance. Negative controls consisted of 0.5 µg of venom samples alone or 5 µg of kininogen alone incubated at 37 °C for 30 min prior to loading. Seven µL of lammeli loading buffer (Bio-Rad, Brisbane, Australia) was added to incubated samples prior to loading into 1 mm tubes;. 12% SDS PAGE gels were prepared using the following recipe: for resolving gel layer, 3.3 mL deionised H_2_O, 2.5 mL 1.5 M Tris-HCl buffer pH 8.8 (Tris—Sigma-Aldrich, St. Louis, MO, USA; HCl—Univar, Wilnecote, UK), 100 μL 10% SDS (Sigma-Aldrich, St. Louis, MO, USA), 4 mL 30% acrylamide mix (Bio-Rad, Hercules, CA, USA), 100 μL 10% APS (Bio-Rad, Hercules, CA, USA), and 4 μL TEMED (Bio-Rad, Hercules, CA, USA); and for the stacking gel layer, 1.4 mL deionised H_2_O, 250 μL 0.5 M Tris-HCl buffer pH 6.8, 20 μL 10% SDS (Sigma-Aldrich, St. Louis, MO, USA), 330 mL 30% acrylamide mix (Bio-Rad, Hercules, CA, USA), 20 μL 10% APS (Bio-Rad, Hercules, CA, USA), and 2 μL TEMED (Bio-Rad, Hercules, CA, USA). The 10 × gel running buffer was prepared using the following recipe: 250 mM Tris (Sigma-Aldrich, St. Louis, MO, USA), 1.92 M glycine (MP Biomedicals), 1% SDS (Sigma-Aldrich, St. Louis, MO, USA), and pH 8.3. Four µL of standard unstained ladder (details) was used as a molecular weight reference on each end of the gels.

### 5.8. Statistical Analyses and Figure Production

All statistical analyses (ANOVA, Student’s *t*-test) and Figures were produced in GraphPad PRISM 8.1.1 (GraphPad Prism Inc., La Jolla, CA, USA).

## Figures and Tables

**Table 1:** Coagulotoxicity protocols.

Factor Inhibition	Methodology Details
FIXa	**Step 1:** 50 μL venom + 50 μL 0.025 M calcium + 25 μL OK buffer + 50 μL PPL + 25 μL Factor IXa (15 μg/mL) (HTI cat. #HCXIA-0160) **Step 2:** 120 s incubation at 37 °C **Step 3:** Addition of 75 μL human plasma
FXIa	**Step 1:** 50 μL venom + 50 μL 0.025 M calcium + 25 μL OK buffer + 50 μL PPL + 25 μL Factor XIa (15 μg/mL) (Haemonetics Technologies Incorporated (HTI) cat. #HCXIA-0160) **Step 2:** 120 s incubation at 37 °C **Step 3:** Addition of 75 μL human plasma
FXa	**Step 1:** 50 μL venom + 50 μL 0.025 M calcium + 25 μL OK buffer + 50 μL PPL + 25 μL Factor Xa **Step 2:** 120 s incubation at 37 °C **Step 3:** Addition of 75 μL human plasma
Thrombin	**Step 1:** 50 μL venom + 50 μL 0.025 M calcium + 25 μL OK buffer + 50 μL PPL + 25 μL thrombin (Stago Liquid Fib kit cat. #00611) **Step 2:** 120 s incubation at 37 °C **Step 3:** Addition of 75 μL human plasma

**Table 2:** Zymogen activation protocols.

Human Zymogen Activation Assays	Methodology Details
Blank wells	20 μL of enzyme buffer without calcium (150 mM NaCl, and 50 mM Tri-HCl (pH 7.3) + 10 μL PPL
FVII, FIX, FX, FXI, FXII, Prothrombin control wells	10 μL of enzyme buffer without calcium (150 mM NaCl, and 50 mM Tri-HCl (pH 7.3) + 10 μL phospholipid (PPL) + 10 μL (10 μg/mL FVII or FIX, FX, FXI, FXII) or 1 μg/mL prothrombin.
FVIIa, FIXa, FXIa, FXIIa, Thrombin control wells	10 μL of enzyme buffer without calcium (150 mM NaCl, and 50 mM Tri-HCl (pH 7.3) + 10 μL PPL + 10 μL (10 μg/mL FVIIa or FIXa, FXa, FXIa)(replaced with 10 μL kaolin (5 mg of kaolin/mL for FXIIa control) or 1 μg/mL Thrombin).
Venom control wells	10 μL of enzyme buffer without calcium (150 mM NaCl, and 50 mM Tri-HCl (pH 7.3) + 10 μL PPL + 10 μL venom (50 μg/mL).
Venom + zymogen wells	10 μL zymogen (10 μg/mL FVII or FIX, FX, FXI, FXII or 1 μg/mL prothrombin) + 10 μL PPL + 10 μL venom (50 μg/mL)

## Data Availability

All data are presented in the graphs.
